# Transplant Biology at a Crossroads: Surgeons can now give patients a new hand or even a new face, but they still can't provide any guarantees that the benefits are worth the risks.

**DOI:** 10.1371/journal.pbio.0060070

**Published:** 2008-03-18

**Authors:** Caitlin Sedwick

## Abstract

Despite major advances in transplantation biology, allowing transplants not just of critical organs like heart and kidney but also of limbs and faces, researchers are still struggling to minimize the risks from achieving the level of immunosuppression needed to make the body accept foreign tissues.

Matt Scott allows that he might not have been thinking too clearly that night in 1985 when the accident happened. After all, it was 2 a.m. when he and his friends returned home from a night of indulging in seasonal cheer, a few days before Christmas. That's when someone thought it might be fun to blow up the old firecracker that had been sitting around the house since midsummer. “I happened to be the one to pick it up,” recalls Scott, then 24 and a paramedic. “I smoked at the time, and I was fooling around with my cigarette, touching the fuse to the tip of my cigarette, never expecting it to light. But it ignited pretty quickly, and burned even more quickly, ” he says. His friend screamed at him to throw it away. “But I just took two steps away from him into the kitchen, and it exploded.”

The radial artery in Scott's left arm was severed, splattering the kitchen wall with blood. Most of his left hand was gone. He clamped down on the injured limb with his other hand, thinking, “My career is over. My life is over. I can't believe I did this.”

Scott and his surgeons decided that what remained of his left hand was not worth saving, so Scott agreed to amputation and was fitted with a prosthesis. As it turned out, neither his career nor his life was over; Scott remained a field paramedic for several years and adapted to the prosthesis. He married and had two sons. But eventually, he came to loathe the artificial hand. “It was a good prosthesis,” he says, “but it was cumbersome, it was uncomfortable, and it wasn't flesh and blood. It became odious to me.”

When he read more than 10 years after his injury about a group of surgeons at Jewish Hospital in Louisville, Kentucky, who were planning to conduct a hand transplant despite widespread concern about the risks, Scott was intrigued and contacted the group. He fit the criteria the surgeons were looking for: he had experienced the loss of his limb some time ago and although he learned to live with the prosthesis, his desire for a flesh-and-blood replacement was strong enough that he was willing to take some significant risks.

## A Risky Proposition

In order to obtain a hand transplant from a genetically dissimilar individual, Scott had to agree to take a lifelong regimen of medications designed to suppress his immune system and prevent rejection of the grafted tissue (see Box 1). But immunosuppressive drugs carry serious risks. They can disrupt immune surveillance—dampening the body's main line of defense against infection and disease—and damage other body organs and systems. Hypertension, infection, tumors, and premature death are just some of the complications that can arise from the level of immunosuppression required for transplantation, says Warren Breidenbach, assistant clinical professor of surgery at the University of Louisville and the head of Scott's transplant team. “The issue is always whether the risk is worth the benefit.”

**Box 1. Wrench in the Works**

Any transplantation procedure between a genetically different donor and recipient carries the near-certainty that the recipient's immune system will relentlessly attack the grafted tissue without the lifelong administration of potentially toxic immunosuppressive drugs. However, it's somewhat ironic that the use of immunosuppressive drugs aims to hamstring a perfectly normal immune system that is doing just what evolution designed it to do: detect and respond to a foreign interloper.

By ignoring normal body structures while responding aggressively to the abnormal or unfamiliar, the immune system retains enough versatility to respond to pathogens or disease states like cancer while ignoring healthy tissues—except in rare cases of autoimmunity where a genetic error or environmental factors cause the immune system to attack the body. This arrangement poses a special challenge for tissue transplantation. That's because although a donated organ, limb, or patch of skin may be absolutely healthy, it can still look like foreign matter to a recipient's immune system if the donor is genetically different.

Of special importance are genetic mismatches between blood groups (A, B, AB, or O) and between the donor's and recipient's complement of major histocompatibility complex (MHC) alleles (gene variants involved in alerting the immune system to the presence of foreign substances). Blood group differences imperil tissue transplantation, because pre-formed antibodies directed against the blood type determinants can provoke immediate and irreversible tissue rejection by attacking the vascular endothelium (called hyperacute rejection). A transplant cannot be performed when the recipient has high levels of antibodies to the donor's blood group. Such blood group incompatibilities may impair the long-term survival of some kinds of organ transplants. Incompatibilities between the recipient's and donor's MHC gene variants can also provoke immune reactions to the graft, ranging from “acute rejection” in a matter of weeks, to “chronic rejection” years after transplantation. If pre-existing antibodies to the donor MHC molecules are detected before a transplant, then transplantation can't be carried out.

Usually only people within the same family—a recipient's twin, sibling, or in rare cases, a parent—are likely to provide a matched set of MHC alleles. However, for most kinds of transplantation (with the prominent exception of bone marrow transplants), immune responses that would otherwise lead to graft rejection can be suppressed by a regimen of powerful immunosuppressive drugs, allowing patients to receive tissue transplants even from genetically different donors with ABO or MHC mismatches.

Such decisions are relatively clear-cut when forgoing a transplant means imminent death, as in the case of a heart transplant patient, or when the alternative means a drastically reduced quality of life, as in the case of a kidney patient who would otherwise face a life-long weekly dialysis regimen. (In such cases, organ availability is a much larger concern than immunosuppression.) The calculation for something like a hand transplant, however, is far more complicated; the procedure can improve quality of life but cannot extend it. “Many patients would rather live with an amputation than accept the risk of a transplant,” says Breidenbach.

Transplantation of a limb or other complex body structure involves moving many different tissues, potentially including nerve, muscle, bone, and skin. Each tissue type might provoke its own immune reaction, explaining why limb transplantation was long considered impossible: the sheer variety of challenges it would present to the recipient's immune system and the potentially deadly levels of immunosuppression needed to overcome them appeared insurmountable.

Solid organ transplants typically involve the transfer of a limited range of different tissue and cell types, but transplantation of a limb or other complex body structure, known as “composite tissue allotransplantation,” involves moving many different tissues, potentially including nerve, muscle, bone, and skin. Each tissue type involved in the transplant might provoke its own immune reaction in a recipient, explaining why limb transplantation was long considered impossible: the sheer variety of challenges it would present to the recipient's immune system and the potentially deadly levels of immunosuppression needed to overcome them appeared insurmountable. It wasn't until the late 1990s that experiments conducted in animal models showed that the procedure could be viable and rejection could be prevented with the right combination of immunosuppressive drugs.

Even so, when Scott was considering his hand transplant, the long-term risks and benefits of the procedure were unknown. When he agreed to have the surgery done in January of 1999, very few humans had undergone the procedure, so no one really knew how long immunosuppression would work, or whether a strong immune response to the graft might eventually overcome the drugs and cause rejection. The first hand transplant patient, a New Zealand man named Clint Hallam, kept his new hand for only about 3 years. For some reason, Hallam ceased taking his immunosuppressive drugs. As a result, he started experiencing rejection and decided to have the transplanted limb amputated. Jean-Michel Dubernard, a clinician at the University of Lyons and head of Hallam's transplant team, said the doctors watched helplessly as the hand's function and appearance degraded. “Even at the time of amputation, pathological studies indicated the rejection may have been reversible if it were treated,” he says.

Yet even with complete compliance, doctors could offer no guarantees. Breidenbach says that in 1998, his team predicted that the odds were 50–50 that a transplanted hand would last 1 year, and 1 in 4 that it would last 5 years. “And we were considered wildly optimistic,” he adds. Nonetheless, Scott, the first patient in the United States to have the procedure done, has lived with his new hand for 9 years without incident, and other efforts have seen similar success. For example, Dubernard's group also worked on the first double hand transplant recipient, whose grafts have survived more than 8 years. By 2005, at least 18 patients worldwide had undergone single or double hand transplantation [1]. In America and Western Europe, the only cases where detailed information is available, no patients lost their transplanted limb as long as they remained compliant with the immunosuppressive drug regimen. Breidenbach says the success of hand transplantation has been “nothing less than shocking” to many in the medical community.

## New Frontiers

Buoyed by the success, transplant doctors have moved on to other kinds of composite tissue transplantation [2] including, famously, the first partial facial tissue transplant in 2004. The patient in that case, Isabelle Dinoire, had suffered severe facial trauma from an attack by her own dog. Dubernard, who treated Dinoire, says the attack destroyed her lips and much of the muscle on one side of her face. She could not speak clearly, could not eat or drink without a gastric tube, and was afraid to go out in public.

A major goal of a facial tissue transplant is to cover the injury, which, depending on the severity or extent of the injury, is difficult to achieve with reconstructive plastic surgery. “In order to recover an entire face, we need about 1,200 square centimeters of skin, and in the most ideal situation, pliable skin, in one piece,” says Maria Siemionow, whose team at the Cleveland Clinic is seeking to perform a similar face transplant procedure in the United States. The body can't supply skin that meets these requirements.

As with hand transplants, facial tissue transplantation can involve the transfer and reattachment of skin, muscle, nerves, and sometimes bone. The recovery of sensory or motor nerve function can spell the difference between expressive and functional transplanted tissue and a mostly cosmetic improvement. In particular, whenever surgeons must reconnect a severed motor nerve, there is a risk of dyskinesia, where impaired motor nerve function prevents patients from controlling the expressivity of the transplanted tissue. These are just some of the issues (to say nothing of the host of psychological considerations) that need to be taken into account when deciding what kinds of injuries should be treated by transplantation.

Dinoire's surgical team faced the considerable technical challenge of restoring motor function as well as sensory function. They took a triangle of skin and muscle including nose and lips from a brain-dead donor and grafted it onto the injured site. Two years after the procedure, Dinoire has recovered enough sensitivity to feel heat, cold, and pressure in the grafted skin. She can also eat and speak more easily. A video accompanying the follow-up report on Dinoire's surgery [3] shows her pronouncing difficult phonemes, like “p” and “f”. Other recipients of composite tissue transplantation have experienced similar success with functional restoration of transplanted tissues. Scott says his transplanted left hand has achieved about 50% of the sensitivity and dexterity that he has in his right hand—a huge improvement over the limited functionality he had with the prosthesis.

Perhaps most importantly for both Scott and Dinoire, they can now go out in public without fear of ridicule. Just knowing that he has a flesh-and-blood hand has made Scott happier and more confident in everyday life. “I was a master of hiding my prosthesis from people, greeting them in such a way that my body language would deflect their attention from looking at it,” he says. But that's all in the past. “I don't know how to explain it—but whatever dark cloud was in my soul is no longer there.”

## Uncertain Future

For Scott, the benefits of his procedure have been worth it, even though he went through some rough patches. He says the first year was very unpleasant as his body adapted to the drugs, initially administered at very high levels. He also experienced two mild rejection episodes during the first year while his doctors worked toward finding the right balance of immunosuppression. During his two rejection episodes, the hand's skin grew red and looked blistered, but overall, the complications Scott experienced were relatively minor.

Others haven't been so lucky. For example, the Louisville group's second hand transplantation patient experienced a condition called avascular necrosis, or bone death, from lost blood supply in the hip as a side-effect of his immunosuppressive drugs. The patient elected for hip-replacement surgery rather than risk rejection of the hand from reducing immunosuppression.

**Figure 1:**
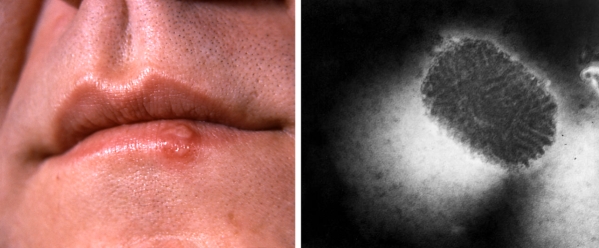
Viral Infections Are a Common Side-Effect of Immunosuppression An oral herpes, or cold sore, infection (causing sores like the one in the image on the left) on transplanted tissue triggered a rejection episode in the first partial face transplant recipient. Later, the patient suffered an outbreak of water warts (which are caused by the poxvirus Molluscum contagiosum, pictured at right) on the transplanted tissue and her own skin as a consequence of the immunosuppressive regimen. (Credits: Herpes image at left courtesy of Dr. Hermann at the Centers for Disease Control and Prevention, Atlanta, Georgia, United States. Molluscum image at right courtesy of Centers for Disease Control and Prevention)

Dinoire also experienced several setbacks during the first year, including two episodes of renal failure caused by her immunosuppressive drugs. Her doctors altered the types and amounts of drugs she was taking, and her kidney function has since recovered. But she's also suffered other complications, including a herpes virus infection on her lips that may have triggered a rejection episode (see Figure 1). After a later rejection episode, she also developed a case of poxvirus infection on her graft and her own facial tissue.

What the future holds for these patients is unknown. Even with far more common and well-understood procedures such as kidney transplants, survival of grafted organs in the long term is threatened by eventual immune rejection. Transplantation specialists are eager to push the boundaries of medicine and science in an effort to improve the chances of graft survival and reduce the toxicity and morbidity of immunosuppressive drugs. While many research groups and pharmaceutical companies focus on developing more-effective and less-toxic medications, others hope to eliminate immunosuppressive therapy altogether—some regard this prospect as the “Holy Grail” of transplantation biology.

“The generalized approach in transplantation for the last 50 years has been what I call ‘blindfolding the immune system,’” explains Jeffrey Bluestone, director of the Immune Tolerance Network. Bluestone says that steroids, calcineurin inhibitors, and similar drugs have all been designed to prevent the immune system from recognizing these foreign components. If immune cells can't recognize a tissue as foreign, they can't destroy it. But he says that the more innovative, and more difficult, strategy involves reeducating the immune system, to somehow convince immune cells that the grafted tissue is not foreign.

In America and Western Europe, the only cases where detailed information is available, no patients have lost their transplanted limb as long as they remained compliant with the immunosuppressive drug regimen. The success of hand transplantation has been “nothing less than shocking” to many in the medical community.

Many transplant groups are pursuing the possibility of persuading the immune system to see the grafted tissue as “self” and ignore it—to create a situation called “immune tolerance.” One current approach springs from experiments in animal models [4] that show that bone marrow from a transplant donor, when transferred to a recipient, can cause the recipient's immune system to ignore the presence of other tissue transplants from the same donor. Such protocols have already been used in kidney transplants, allowing doctors to withdraw immunosuppression for some patients and yet retain functioning grafts [5,6], though many questions remain about potential side effects and the long-term effectiveness of this strategy. Dubernard's group also tried a related approach with Dinoire's transplant by pre-administering bone marrow–derived stem cells from the same donor who supplied the facial tissue. However, it is difficult to tell whether there was any effect; Dinoire still experienced two episodes of acute rejection. Some variations of this procedure also carry a risk that elements of the transplanted bone marrow may attack the recipient—a life-threatening complication termed “graft versus host disease.”

Transplantation specialists are eager to push the boundaries of medicine and science in an effort to improve the chances of graft survival and reduce the toxicity and morbidity of immunosuppressive drugs. Many research groups and pharmaceutical companies focus on developing more effective and less toxic medications; others hope to eliminate immunosuppressive therapy altogether—some regard this prospect as the “Holy Grail” of transplantation biology.

Another possible approach involves using new drugs to strengthen existing immune pathways, such as those that regulate inappropriate activity, to tamp down immune responses. For example, some groups are testing antibodies or chimeric molecules that bind certain cell surface molecules that transmit “shut-down” signals to immune cells. These signals cause immune cells to become dormant or to undergo cellular suicide, thus limiting an inappropriate (autoimmune) or undesired (transplant rejection) immune response [7].

Still another approach involves bolstering a population of regulatory immune cells that limit the intensity or duration of an immune response. For example, Bluestone's research group is working with an antibody called anti-CD3 that appears to promote expansion of this regulatory cell population. This compound is already being tested in transplant settings. If it proves possible to direct the efforts of the regulatory immune cells specifically toward protecting transplanted tissue, clinicians could potentially prevent the rejection process from getting started—thus removing the need for immunosuppressive drugs by priming the patient's own regulatory immune cells to protect the graft. However, such work is still in its early stages and few trials have been attempted in human subjects.

Philip Halloran, a prominent researcher on kidney transplantation at the University of Alberta, feels that none of the new approaches aimed at inducing immune tolerance are ready for clinical trials that involve the withdrawal of immunosuppression. For one thing, he worries that many of the protocols may not adequately target the antibody-mediated pathways that cause the kind of chronic rejection that is observed in kidney transplants [8]. Additionally, he feels that other experimental protocols—particularly those involving bone marrow transplants—may pose unnecessary risks to patients. With so much yet to learn about the immunological pathways involved in rejection, Halloran argues that it's too hard to predict whether individual patients might benefit from such experimental protocols and it's too risky to take transplant patients off immunosuppressive drugs. “How many of those patients will be able to go off immunosuppression and how many of them will undergo severe rejection to find out they can't come off immunosuppression? That's very much an open question.”

In the end, it may prove impossible to completely eliminate risks to patients when it comes to something as complex as transplantation. However, regardless of the risks, someone will always be willing to push the boundaries in medicine and science. And patients like Scott and Dinoire—and thousands of organ transplant patients—clearly demonstrate the need to do so. Although far fewer patients will benefit from investments in transplant research than, say, cancer or cardiac-related diseases, Halloran makes the case that money invested in transplantation is money well spent. “It's a lightweight in terms of patient numbers but a heavyweight in terms of the insights it generates for the rest of medicine.”

For more information on transplantation, go to http://handtransplant.org/, http://immunetolerance.org/, or http://www.kidney.org/.
